# Valores de Referência em Ressonância Magnética Cardiovascular: Entre a Validação Local e a Harmonização Global

**DOI:** 10.36660/abc.2026458

**Published:** 2026-07-24

**Authors:** Miguel Correia

**Affiliations:** 1 Centro Hospitalar Tondela Viseu Viseu Portugal Centro Hospitalar Tondela Viseu, Viseu – Portugal; 2 Hospital CUF Viseu Viseu Portugal Hospital CUF Viseu, Viseu – Portugal

**Keywords:** Imageamento por Ressonância Magnética, Valores de Referência, Padrões de Referência

A ressonância magnética cardiovascular (RMC) se consolidou nas últimas décadas como o método de referência para avaliação morfofuncional cardíaca, permitindo a quantificação precisa de volumes ventriculares, fração de ejeção e massa miocárdica, além da caracterização tecidual de múltiplas cardiopatias.^1-4^ A elevada reprodutibilidade das suas medições constitui uma das principais vantagens em relação a outras modalidades de imagem, tornando a RMC uma ferramenta essencial tanto na prática clínica quanto na investigação cardiovascular.^2,3^

A interpretação adequada dos parâmetros obtidos por RMC depende, contudo, da existência de valores de referência robustos e aplicáveis à população estudada. Neste contexto, o artigo "Valores de Normalidade para Ressonância Magnética Cardiovascular uma Revisão Analítico-Comparativa com Ênfase na População Brasileira", publicado nesta edição dos Arquivos Brasileiros de Cardiologia,^5^ aborda uma questão particularmente relevante para a cardiologia brasileira: a aplicabilidade dos valores normativos internacionais à realidade nacional.

Os autores realizaram uma análise comparativa entre os valores de referência obtidos em uma coorte brasileira saudável descrita por Macedo et al.^6^ e os valores derivados da mais recente atualização das *Guidelines* da *Society for Cardiovascular Magnetic Resonance* (SCMR).^7^ A principal conclusão do estudo foi a existência de elevada concordância global entre os parâmetros morfofuncionais avaliados, com diferenças estatisticamente significativas restritas a um número reduzido de variáveis, correspondendo essencialmente aos diâmetros ventriculares esquerdos e à massa ventricular esquerda.^5^

Estes resultados merecem destaque. Historicamente, a necessidade de estabelecer valores normativos específicos para diferentes populações tem sido frequentemente defendida com base em diferenças antropométricas, étnicas e ambientais. No Brasil, país marcado por grande diversidade genética e heterogeneidade regional, essa preocupação parece particularmente justificável. No entanto, os dados apresentados sugerem que, quando adequadamente indexados à superfície corporal, os principais parâmetros cardíacos obtidos por RMC apresentam comportamento notavelmente semelhante ao observado em grandes coortes internacionais.^7-10^

Tais resultados reforçam uma mensagem importante: parte substancial da variabilidade observada entre estudos provavelmente decorre mais de diferenças metodológicas do que de verdadeiras diferenças biológicas entre populações. Aspectos metodológicos aparentemente simples, como a inclusão ou exclusão dos músculos papilares, os critérios de delimitação endocárdica e epicárdica, os softwares utilizados para pós-processamento ou mesmo os planos empregados para determinadas medições, podem influenciar significativamente os valores obtidos.^1,2,8^

Nesse sentido, não surpreende que as maiores divergências identificadas pelos autores tenham ocorrido justamente em parâmetros particularmente sensíveis à metodologia de aquisição e análise. O próprio estudo destaca que os diâmetros ventriculares esquerdos foram obtidos por técnicas distintas nas publicações comparadas, o que provavelmente explica parte das diferenças observadas.^5^ Esta constatação reforça que a harmonização dos protocolos de aquisição e análise pode ser tão importante quanto a definição dos próprios intervalos de normalidade.

Outro mérito do trabalho reside em destacar a evolução das bases internacionais de referência. As atualizações recentes da SCMR incorporaram dados provenientes de múltiplas coortes internacionais, incluindo a população brasileira estudada por Macedo et al., ampliando significativamente a robustez estatística dos intervalos de referência atualmente disponíveis.^6,7^ A utilização de grandes bases multicêntricas e multiétnicas representa um avanço importante para a construção de modelos normativos mais abrangentes e potencialmente mais aplicáveis em diferentes cenários clínicos.

O manuscrito apresenta ainda uma proposta pragmática para gradação da anormalidade baseada em múltiplos do desvio-padrão da média. Embora esta abordagem não faça parte das recomendações formais da SCMR,^7^ ela procura responder a uma necessidade frequentemente encontrada na prática clínica: a comunicação objetiva da magnitude das alterações observadas. A utilização exploratória de limiares correspondentes a 1.5, 3 e 4.5 desvios-padrão para caracterizar alterações ligeiras, moderadas e severas, respetivamente, constitui uma proposta interessante que poderá estimular futuras investigações e processos de validação.^5^

Importa, contudo, interpretar os resultados com cautela. Como reconhecido pelos autores, a comparação foi realizada a partir de dados sumarizados, sem acesso aos microdados das coortes originais.^5^ Tal limitação impede ajustes individuais para variáveis potencialmente relevantes, como idade, superfície corporal e composição étnica, além de impossibilitar análises mais sofisticadas de equivalência populacional. Consequentemente, os achados devem ser interpretados como evidência de concordância global e não como validação formal dos valores internacionais para a população brasileira.

Apesar dessas limitações, o estudo fornece uma contribuição relevante para a imagem cardiovascular nacional. Seus resultados sugerem que os valores de referência internacionalmente recomendados podem ser utilizados na prática clínica brasileira, desde que respeitadas as particularidades metodológicas e interpretativas de cada contexto.^5^ Mais importante ainda, o trabalho desloca o foco do debate da busca por diferenças populacionais para a necessidade de maior padronização metodológica.

Numa era caracterizada pela crescente integração científica internacional e pela expansão das ferramentas automatizadas de análise de imagem, a questão central talvez não seja determinar se os valores de normalidade devem ser brasileiros ou internacionais, mas assegurar que sejam obtidos por métodos robustos, reproduzíveis e clinicamente relevantes. Sob esta perspetiva, o estudo comentado representa um passo importante na construção de uma linguagem comum para a RMC, favorecendo a harmonização da prática clínica e a comparabilidade dos resultados entre centros nacionais e internacionais.
