# Supplementary material for: Valores de Referência em Ressonância Magnética Cardiovascular: Entre a Validação Local e a Harmonização Global
Source: Arq Bras Cardiol. 2026 Jul 24;123(6):e20260458. [Article in Portuguese] doi: 10.36660/abc.2026458 (PMC13668452; doi:10.36660/abc.2026458)
Supplement: Translation

# Translation: Reference Values in Cardiovascular Magnetic Resonance Imaging: Between Local Validation and Global Harmonization

**DOI:** 10.36660/abc.2026458i

**Authors:** Miguel Correia

**Affiliations:** 1 Viseu Portugal Centro Hospitalar Tondela Viseu, Viseu – Portugal; 2 Viseu Portugal Hospital CUF Viseu, Viseu – Portugal

**Keywords:** Magnetic Resonance Imaging, Reference Values, Reference Standards

Cardiovascular magnetic resonance imaging (CMR) has become established in recent decades as the gold standard method for cardiac morphofunctional assessment, allowing for the precise quantification of ventricular volumes, ejection fraction, and myocardial mass, as well as the tissue characterization of multiple heart diseases.^1-4^ The high reproducibility of its measurements is one of its main advantages over other imaging modalities, making CMR an essential tool in both clinical practice and cardiovascular research.^2,3^

However, the proper interpretation of parameters obtained by CMR depends on the existence of robust reference values applicable to the studied population. In this context, the article "Normal Reference Values for Cardiovascular Magnetic Resonance: An Analytical–Comparative Review with Emphasis on the Brazilian Population", published in this issue of the *Arquivos Brasileiros de Cardiologia*,^5^ addresses a question that is particularly relevant to Brazilian cardiology: the applicability of international normative values to the national reality.

The authors performed a comparative analysis between reference values obtained in a healthy Brazilian cohort described by Macedo et al.^6^ and values derived from the most recent update of the Society for Cardiovascular Magnetic Resonance (SCMR) Guidelines.^7^ The main conclusion of the study was the existence of high overall agreement between the morphofunctional parameters evaluated, with statistically significant differences restricted to a small number of variables, essentially corresponding to left ventricular diameters and left ventricular mass.^5^

These results deserve highlighting. Historically, the need to establish specific normative values for different populations has often been advocated based on anthropometric, ethnic, and environmental differences. In Brazil, a country marked by great genetic diversity and regional heterogeneity, this concern seems particularly justified. However, the data presented suggest that, when adequately indexed to body surface area, the main cardiac parameters obtained by CMR exhibit remarkably similar behavior to that observed in large international cohorts.^7-10^

These results reinforce an important message: a substantial part of the variability observed between studies likely stems more from methodological differences than from true biological differences between populations. Seemingly simple methodological aspects, such as the inclusion or exclusion of papillary muscles, the criteria for endocardial and epicardial delimitation, the software used for post-processing, or even the planes employed for certain measurements, can significantly influence the values obtained.^1,2,8^

In this sense, it is not surprising that the greatest divergences identified by the authors occurred precisely in parameters particularly sensitive to the acquisition and analysis methodology. The study itself highlights that left ventricular diameters were obtained using different techniques in the compared publications, which probably explains some of the observed differences.^5^ This finding reinforces that the harmonization of acquisition and analysis protocols can be as important as the definition of the normal ranges themselves.

Another merit of the work lies in highlighting the evolution of international reference databases. Recent updates to the SCMR have incorporated data from multiple international cohorts, including the Brazilian population studied by Macedo et al., significantly increasing the statistical robustness of the currently available reference ranges.^6,7^ The use of large multicenter and multiethnic databases represents an important advance in the construction of more comprehensive and potentially more applicable normative models in different clinical scenarios.

The manuscript also presents a pragmatic proposal for grading abnormality based on multiples of the standard deviation from the mean. Although this approach is not part of the formal recommendations of the SCMR,^7^ it seeks to address a need frequently encountered in clinical practice: the objective communication of the magnitude of the observed changes. The exploratory use of thresholds corresponding to 1.5, 3, and 4.5 standard deviations to characterize mild, moderate, and severe changes, respectively, constitutes an interesting proposal that may stimulate future research and validation processes.^5^

However, the results should be interpreted with caution. As acknowledged by the authors, the comparison was made using summarized data, without access to the microdata of the original cohorts.^5^ This limitation prevents individual adjustments for potentially relevant variables, such as age, body surface area, and ethnic composition, and also makes more sophisticated population equivalence analyses impossible. Consequently, the findings should be interpreted as evidence of overall agreement and not as formal validation of international values for the Brazilian population.

Despite these limitations, the study provides a relevant contribution to national cardiovascular imaging. Its results suggest that internationally recommended reference values can be used in Brazilian clinical practice, provided that the methodological and interpretative particularities of each context are respected.^5^ More importantly, the work shifts the focus of the debate from the search for population differences to the need for greater methodological standardization.

In an era characterized by increasing international scientific integration and the expansion of automated image analysis tools, the central question may not be whether normality values should be Brazilian or international, but rather ensuring that they are obtained through robust, reproducible, and clinically relevant methods. From this perspective, the study discussed represents an important step in building a common language for CMR, promoting the harmonization of clinical practice and the comparability of results between national and international centers.
